# MicroRNA-33b inhibits cell proliferation and glycolysis by targeting hypoxia-inducible factor-1α in malignant melanoma

**DOI:** 10.3892/etm.2021.10729

**Published:** 2021-09-13

**Authors:** Yue Zhao, Cuiling Wu, Lina Li

Exp Ther Med 14:1299–1306, 2017; DOI: 10.3892/etm.2017.4702

Following the publication of this paper, it was drawn to the Editors’ attention by a concerned reader that the western blotting assay data shown in Figs. 5 and 6 were strikingly similar to data appearing in different form in other articles by different authors. Owing to the fact that the contentious data in the above article had already been published elsewhere, or were already under consideration for publication, prior to its submission to *Experimental and Therapeutic Medicine*, the Editor has decided that this paper should be retracted from the Journal. After having been in contact with the authors, they agreed with the decision to retract the paper. The Editor apologizes to the readership for any inconvenience caused.

